# Obstructive sleep apnoea patients vs laryngopharyngeal reflux disease: Non-invasive evaluation with NBI and pepsin detection in tears

**DOI:** 10.17305/bjbms.2021.6712

**Published:** 2022-02-11

**Authors:** Annalisa Pace, Valeria Rossetti, Alessandro Milani, Giannicola Iannella, Salvatore Cocuzza, Antonino Maniaci, Danilo Alunni Fegatelli, Annarita Vestri, Antonio Greco, Marco de Vincentiis, Francesca Giovannetti, Rocco Plateroti, Giuseppe Magliulo

**Affiliations:** 1 Department of Scienze Chirurgiche, Sapienza University of Rome, Rome, Italy; 2 Department of Organi di Senso, Sapienza University of Rome, Rome, Italy; 3 Department of Otolaryngology, Head-Neck and Oral Surgery Unit; Department of Head-Neck Surgery, Morgagni Pierantoni Hospital, Forlì, Italy; 4 Department of Otorinolaringoiatria, University of Catania, Catania, Italy; 5 Department of Public Health and Infectious Diseases, Sapienza University of Rome, Rome, Italy

**Keywords:** Laryngopharyngeal reflux disease, narrowband imaging, obstructive sleep apnea, pepsin

## Abstract

Obstructive sleep apnea (OSA) and laryngopharyngeal reflux disease (LPR) are two common diseases that lower patients’ quality of life. OSA is defined by cyclic events of airflow obstruction that occurs during sleep, while LPR is characterized by upper airway inflammatory signs and symptoms due to the return of gastroduodenal gaseous and liquid elements. pH-metry is the gold standard in LPR diagnosis, but considering its invasiveness among other negative traits, questionnaires that catalog symptoms and signs of the disease such as reflux symptom index (RSI) and reflux finding score (RFS) are preferred. Moreover, LPR can be evaluated by testing the presence of pepsin in tears, and narrowband imaging (NBI) has been introduced for the early diagnosis of larynx oncological disease. This paper aims to test whether LPR is more frequent in OSA patients than in control ones, performing a non-invasive protocol composed of RSI and RFS test (with light vs. NBI techniques) followed by pepsin detection in tears. Sixty-eight LPR patients were enrolled in the study (45 with OSA and 23 without OSA). A strong linear relationship between apnea-hypopnea index (AHI) and oxygen desaturation index (ODI) was found, and patients who presented pepsin in tears had higher values of AHI and ODI in comparison to patients without it. Pathological RFS and NBI showed higher values of AHI and ODI in comparison to the control group. Furthermore, pathological RSI showed higher values of AHI and ODI in comparison to the control group. In conclusion, this diagnostic combined non-invasive protocol may be a good method to perform an early diagnosis of LPR.

## INTRODUCTION

Obstructive sleep apnea (OSA) is a disease defined by cyclic events of airflow obstruction that occurs during sleep, due to the collapse of an upper airway structure. OSA is frequent in the general population, with an incidence of 5% in adult subjects [1].

Over the past years, great importance has been given to the complications associated with OSA. Cardiovascular, metabolic, and neurological ones are given the highest consideration, but it is important to remember all the others, less severe, that reduce patients’ quality of life [2-3].

Laryngopharyngeal reflux disease (LPR) is one of the most common diseases reported in the general population characterized by upper airway inflammatory signs and symptoms due to the return of gastroduodenal gaseous and liquid elements. Many studies have attempted to explain their connection with OSA [1,4].

The gold standard for LPR diagnosis is pH-metry, but it is invasive, expensive, and unpleasant for the patients. Moreover, this specific type of reflux might not give relevant episodes in the course of 24 hour examination, since LPR is multifactorial and dependent on diet, stress, lifestyle, and drug therapy [5].

Therefore, various questionnaires that catalog symptoms and signs of the disease have been proposed in the literature as alternative and less invasive diagnostic methods, with easiest and cheapest of them being reflux symptom index (RSI) and reflux finding score (RFS) [6]. The latter, in particular, estimates the severity of LPR on larynx lesions during endoscopic evaluation.

At present, in oncological diseases, narrowband imaging (NBI) has been introduced for an early diagnosis, but it has also been described as useful for classifying benign lesions. Galli et al. studied NBI for rhinopharyngolaryngeal reflux in pediatric patients and observed that NBI provides evidence of LPR signs not detected using white light [7]. In 2020, our group performed work to evaluate the possible use of NBI for an integrated endoscopic examination in LPR diagnosis in adult patients. We showed that NBI laryngoscopy was able to recognize 23% more patients with LPR signs than the white light method [8].

However, these methods remain partially objective and operator dependent. It is known that pepsin is a damaging substance that causes LPR lesions. Therefore, the detection of pepsin in the upper airway tract represents a valid and non-invasive diagnostic method [9,10]. In particular, some articles have proposed the evaluation of pepsin tears as a diagnostic, economic, and low invasive method to perform LPR diagnosis. It was proved that LPR patients presented high levels of pepsin concentration in tears in comparison to healthy subjects [10].

The purpose of this pilot study was to test if LPR is more frequent in OSA patients than in control ones, performing a non-invasive protocol composed of RSI and RFS test (with light vs. NBI techniques), associated with pepsin detection in tears.

## MATERIALS AND METHODS

This prospective pilot study was conducted at the “Organi di Senso” Department of “Sapienza” University of Rome from March 2020 to March 2021.

Subjects enrolled were older than 18 years with a suspicion of OSA. Exclusion criteria were: <18 years old, the existence of the oral or laryngeal disease, administration of pump inhibitors or medications for LPR treatment concurrently and 3 months before the study, and subjects that performed domiciliary therapy for OSA.

Patient data were collected in a database: Sex, height, weight, body mass index (BMI), and age. All patients underwent ENT examination and polysomnography (PSG) type III for one night, according to the American Association Sleep Medicine (AASM) 2017 classification [11]. Some authors that tested the accuracy of PSG type III showed a strong correlation between the resulting AHI values and PSG type I [12-15].

Report of the device collected: Time of sleep, respiratory movement of the thorax and abdomen, respiratory airflow, heart rate, arterial oxygen saturation, and patient position. The data were also analyzed following AASM classification. Apnea was characterized by a reduction of airflow not inferior to 90% and a duration of 10 seconds or longer.

Hypopnea was considered in a case of a 30% airflow reduction with a duration inferior to 10 seconds and related arousal, or a 3% O2 saturation drop [15-17]. Apnea-hypopnea index (AHI) was estimated by the mean number of apnea and hypopnea for an hour of sleep.

Two different authors (AP and VR) checked and reviewed all recordings, and a third author (AM) performed random quality checks. Each PSG recording provided a considerable amount of data regarding AHI (supine and not supine); oxygen desaturation index (ODI; supine and not supine); and mean SpO2 (supine and not supine). Finally, AHI (mean between supine and not supine) was considered as the index of severity of OSA disease, following AASM criteria.

As a control group patients, those with an AHI value <5/h were considered. On the contrary, OSA patients were classified into three groups: Mild OSA (AHI 5 and <15), moderate OSA (AHI 15 and <30), and severe OSA (AHI >30).

All patients were analyzed for LPR signs and symptoms using two questionnaires (RSI and RFS). RSI [18] is a self-conducted questionnaire composed of nine questions. Each question goes from 0 to 5 points with a total possible value of 45 points. High suspicion of LPR is related to a score of 13 or more. RFS [19] is based on the evidence and signs of LPR visualized with a fibrolaryngoscope. It takes into account eight findings with a scale that goes from 0 to 26. Endoscopic evaluation of LPR and RFS scores was conducted by a single author with a flexible endoscope joined to a camera and a high-definition monitor (Full HD). LPR is considered present with a score ≥ of 7.

According to a preliminary study conducted by our group [8], NBI may be considered a useful method for LPR diagnosis. Therefore, the calculation of RFS was also based on NBI signs. Moreover, we also employed the new NBI score proposed (Table 1). The maximum total NBI score was 13. NBI grading score of LPR included:

**TABLE 1:**
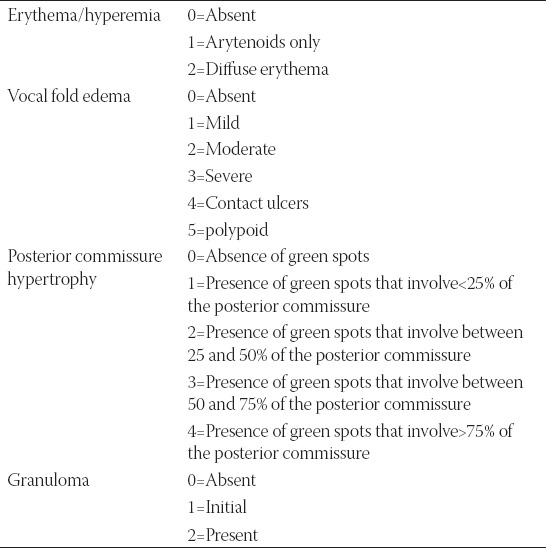
NBI score

- Grade 1 (0–2 points) = Absent or Mild LPR
- Grade 2 (3–7 points) = Moderate LPR
- Grade 3 (8–13 points) = Severe LPR

Endoscopic evaluation with white light was always performed by the same author (VR) adequately trained before the study. On the other hand, the NBI test was conducted by another trained operator (AP) who did not have any information regarding RFS white light results.

Finally, a tear sample for pepsin determination was collected from all patients in the study. The tears were taken early in the morning with a micropipette, a silicone tube with a diameter of 0.3 cm, 2 cm long, diagonally sectioned at 45°, attached to a small vat (diameter of 0,5 cm, 2 cm long), supplied with an aspiration pipe. The micropipette operates by pipetting the tears from the tear lake, located on the bulbar conjunctiva, at the level of the inner chant of the eyelid and depositing the liquid on a glove slide. The collection takes place through the micropipette with a rapid movement, to avoid any subsequent tearing. The tears of both eyes were collected and conveyed into a unique pipe.

Tears sample underwent Peptest™ kit (BIOHIT HealthCare) that determines pepsin quality and quantity in body secretion [9-10]. The test required 100 ml of tears with the addition of 100 ml of 0.01 M citric acid. Each sample was centrifuged at 4000 rpm for 5 minutes. Subsequently, 80 ml of supernatant was collected and added to 240 ml of migration buffer and the mixture was vortexed for 10 seconds. A 80 ml of this mixture was pipetted into the well of the Peptest^TM^ Lateral Flow Device and the results were ready after 15 minutes.

The evaluation is based on the antigen-antibody reaction that uses a monoclonal anti-pepsin antibody (T band reveals the pepsin presence). The system’s integrity is checked by an inner reaction (C band) that validates the directly proportional test (IC and C band). T band, instead, results from pepsin with an intensity directly proportional to its concentration. The kit has a Peptest Cube composed of a display that shows ng/ml of pepsin in fluid in 3 seconds (minimum concentration 16 ng/ml). The results can be negative (only the IC is present), positive (the T and C bands are present), and null (absence of IC signal) [8,9,20].

### Ethical statement

This study was authorized by Sapienza ethical committee (RIF.CE.4841) following the principles of the Declaration of Helsinki. Informed consent was signed by each patient enrolled in the study.

### Statistical analysis

Descriptive statistics were performed to sum up patients’ features: Numerical data were reported as mean (standard deviation) and categorical data as frequency (percentage). A scatter plot was used to describe the relationship between AHI and ODI, and the strength of the linear relationship was assessed using Pearson’s correlation coefficient. Box plots and non-parametric Wilcoxon rank-sum tests were applied for group comparison. Multivariable logistic regression was performed to evaluate the impact of RSI and the presence of pepsin on the grade of OSA adjusted for age and BMI. A value of 0.05 was used as the cutoff for determining statistical significance. All the statistical analyses were performed by the open-source statistical software R (version 4.0.4).

## RESULTS

Sixty-eight consecutive patients, a convenience sample, were enrolled (52 males, M and 16 females, F) of whom 45 (35 M and 10 F) had a diagnosis of OSA and were included in the “case group.” On the contrary, 23 patients (17 M and 6 F) without OSA were enrolled in the “control group.” The data collected regarding weight, height, BMI, and anatomic characteristics of patients (septal nose deviation, turbinate hypertrophy, Mallampati, and Friedman) are reported in Table 2.

**TABLE 2:**
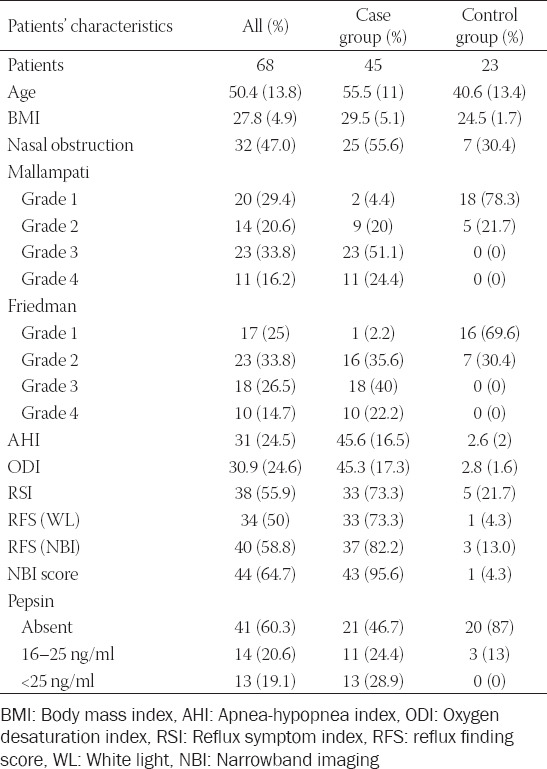
Patients characteristics reported as mean and percentages

**FIGURE 1:**
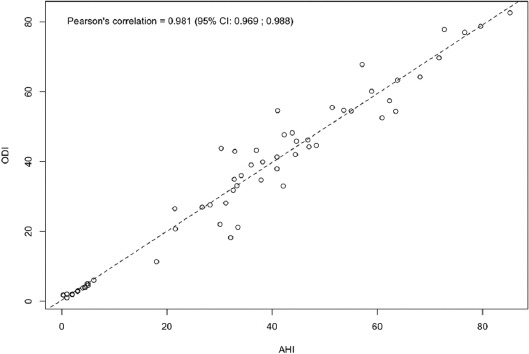
Scatter plot showing apnea-hypopnea index and oxygen desaturation index linear relationship (Pearson’s correlation coefficient).

The scatter plot and the corresponding Pearson’s correlation coefficient showed a strong linear relationship between AHI and ODI (Figure 1). The value of ODI and AHI is reported in Table 2.

**FIGURE 2:**
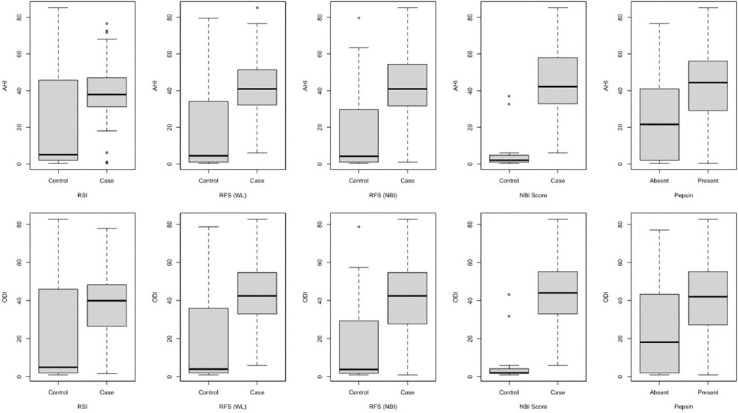
Box plots of apnea-hypopnea index and oxygen desaturation index according to the case and control group.

Pathological RFS and NBI showed higher values of AHI and ODI in comparison to the control group (all *p* < 0.001 are in Figure 2). Pathological RSI also showed higher values of AHI (*p* < 0.006) and ODI (*p* > 0.007) in comparison to the control group, as shown in Figure 2.

Patients who presented pepsin in tears had higher values of AHI and ODI in comparison to patients without it (*p* < 0.001) (Figure 2). There was no association between the presence of pepsin and nasal obstruction (*p* = 0.324).

The comparison between RSI and the presence of pepsin in both groups, corrected for age and BMI, resulted statistically different (*p* < 0.05). The same was found for the comparison between RFS, tested both with white light and NBI, after age and BMI correction (*p* < 0.05).

## DISCUSSION

OSA is a disorder with an elevated incidence in the global population and its consequences have a strong effect on patients’ quality of life. The association with LPR has been confirmed by many studies and different theories are reported regarding its possible etiopathogenesis [1,21-22].

The first hypothesis defined LPR as dependent on increased negative intrathoracic pressure. The latter is consequent to the major respiratory effort, due to the upper airway obstruction, which may also act on the upper esophageal sphincter, reducing the efficacy of its function.

An opposite theory reported that OSA could be worsened by LPR status. Chronic inflammatory status, induced by LPR in the larynx and pharynx, may provoke hypertrophy and thickening of the tissues linked with a sensory impairment and a loss of reflex activity of the larynx during the night [1,23,24]. Moreover, OSA and LPR were evaluated as possible associated to the production of chronic cough. A recent study found that patient with OSA and concomitant LPR is in dependence to OSA’s severity [25].

Furthermore, it should be considered that a characteristic of most OSA patients is a high BMI level. Obese patients present an increased intra-abdominal pressure that reduces the thoracic excursion facilitating OSA and also reduces stomach clearance and increases reflux episodes.

At present, the gold standard for diagnosing LPR is 24 hours pH impedance monitoring. This test is expensive and uncomfortable for the patient. Moreover, a recent review of the literature highlights how this method is useful in the case of gastroduodenal acid fluid reflux while failing to detect non-acid reflux. Its false-negative rate is about 20-50% due to the multifactorial condition of LPR: Lifestyle, diet, and stress. Finally, there is no standardization in terms of probe position and diagnostic criteria [5].

Therefore, in daily clinical practice, the diagnosis of LPR is indirectly conducted by performing validated questionnaires during ENT evaluation. Both RSI and RFS scores were produced by Belafsky [18,19]. RFS, in particular, is a clinical severity score used to classify common laryngoscopic evidence.

We have chosen to perform RSI and RFS since it was a preliminary study and both of them are internationally approved in literature, easier to administer and cheaper [15-16]. RSI was defined as easy administration and highly reproducible method, exhibiting excellent clinical validity. RFS, according to Belafsky et al. [15-16], evidenced an improvement during follow-up of LPR patients treated with PPI therapy with an excellent inter- and intra-observer reproducibility.

However, in the past years, NBI endoscopic evaluation has been performed not only in the oncologic ENT field but also for the study of benign ENT lesions. Galli et al. analyzed the effect of LPR in children using NBI to study cobblestone aspects of the hypopharyngeal mucosa, phlogosis of the tonsillar crypts and adenoid surface, hyperemia, and hypervascularization of subglottic and tracheal mucosa [7].

In 2020, Pace et al. [8] performed a study to evaluate the comparison between LPR studied with white light endoscope and NBI ones. Moreover, they tried to define an NBI classification system to classify the grade of LPR disease. Results showed that RSF assessed by white light examination recognized LPR signs in 65% of the patients, while the same score calculated with NBI laryngoscopy recognized 88% of patients affected by LPR disease. This information testified to the greater sensitivity of NBI laryngoscopy in LPR diagnosis in comparison to the white light one.

For this reason, one of the aims of the current study was to test the frequency of LPR in OSA subjects through the most common questionnaires and testing RFS with white light and NBI. Results agree with the literature related to the increased LPR in OSA subjects tested with both techniques.

The statistical analysis conducted showed that RSI and RFS, tested both with light and NBI, were associated with high values of AHI and ODI. Therefore, symptomatology and symptoms confirmed an elevated presence of LPR in OSA patients.

However, these questionnaires are partly objective since they depend on the patient’s answers and sensation, and the training of the endoscopic operator. One of the authors (VR) was trained to perform a good diagnosis with light. To get more objective results, another author (AP) performed NBI evaluation without knowing the results of white light RFS.

A new non-invasive diagnostic method to study LPR is characterized by the analysis of pepsin in the upper respiratory tract [26]. Many authors researched pepsin in saliva comparing results with 24 hours MII pH monitoring tests result [22-25].

In 2019, Iannella et al. examined LPR in OSA subjects based on salivary pepsin concentration [9]. Finally, they correlated results with the grade of OSA disease. The presence of salivary pepsin was reported in many subjects with sleep apnea even if no correlation was found with the grade of disease.

In the upper respiratory tract, pepsin was described in the tears sample as well. Iannella et al. studied the relationship between LPRD and the concentration level of pepsin in the tears of 20 children affected by LPRD. These children underwent RSI testing and 24 hours MII pH. The percentage of human pepsin in tears, tested with enzyme-linked immunosorbent assay (ELISA), was present in 20% of children affected by LPRD in concentration levels of 3.5, 5.4, 4.0, and 4.2 ng/ml, respectively. There was no pepsin found in the tears of the control group. The presence of pepsin in tears was not correlated to the total number and types of reflux episodes [25].

The first study on pepsin detection in the adult population was conducted by Magliulo et al. [10] in 2020, where results showed that 64% of the LPRD selected patients presented high levels of pepsin concentration in their eyes, while no patients in the control group presented pepsin in tear samples obtained. In this study, a similar result was obtained with 72% of patients with OSA, pathological RSI and RFS score, and with the presence of pepsin in tears. They hypothesized that pepsin reaches pre-corneal tears film passing through the nasolacrimal duct during reflux attacks [10]. However, other possible causes may be pepsin arrival from blood and lacrimal glands cells production. Therefore, it was confirmed that pepsin may be economical and easy to administer method for the LPR presence determination.

## CONCLUSION

Limitations of this study were the small sample size and the lack of MCII as the gold standard in the LPR diagnosis. However, the analysis performed is cheaper and easier to find in the ENT department and the common clinical practice. The diagnostic protocol may be considered as a potential first screening for LPR, avoiding the unuseful and expensive MCII. In this paper, in fact, we did not test any formal hypothesis, but wanted to explore the possible associations between tears and reflux to be able to design an adequately powered diagnostic study. Therefore, further studies, with sample size calculated with statistical power and significance level, are underway to confirm our results.
